# Clonal Variation in Growth Plasticity within a *Bosmina* *longirostris* Population: The Potential for Resistance to Toxic Cyanobacteria

**DOI:** 10.1371/journal.pone.0073540

**Published:** 2013-09-09

**Authors:** Xiaodong Jiang, Qingmei Li, Huishuang Liang, Shiye Zhao, Lihua Zhang, Yunlong Zhao, Liqiao Chen, Wei Yang, Xingyu Xiang

**Affiliations:** School of Life Science, East China Normal University, Shanghai, China; Federal University of Rio de Janeiro, Brazil

## Abstract

Many aquatic organisms respond phenotypically, through morphological, behavioral, and physiological plasticity, to environmental changes. The small-size cladoceran *Bosmina* *longirostris* , a dominant zooplankter in eutrophic waters, displayed reduced growth rates in response to the presence of a toxic cyanobacterium, *Microcystis* *aeruginosa* , in their diets. The magnitude of growth reduction differed among 15 clones recently isolated from a single population. A significant interaction between clone and food type indicated a genetic basis for the difference in growth plasticity. The variation in phenotypic plasticity was visualized by plotting reaction norms with two diets. The resistance of each clone to dietary cyanobacteria was measured as the relative change in growth rates on the “poor” diet compared with the “good” diet. The enhanced resistance to *M* *. aeruginosa* in *B* *. longirostris* was derived from both the reduced slope of reaction norms and the increased mean growth rates with two diets. The large clonal variation within a *B* *. longirostris* population may contribute to local adaptation to toxic cyanobacteria and influence ecosystem function via clonal succession.

## Introduction

Phenotypic plasticity refers to the phenomenon that a genotype produces distinct phenotypes when living in different environmental conditions [1,2]. It is now clear that phenotypic plasticity is widespread in organisms, and mainly involves ecologically relevant morphological, physiological, behavioral and life-history traits [3]. Therefore, plasticity shapes various interactions between organisms and their abiotic and biotic environments, alters ecological patterns and processes at diverse levels, and even affects evolutionary trajectory [1,3].

Phenotypic plasticity can be considered as an omnipresent part of organisms [2]. As a property of a genotype, phenotypic plasticity can be adaptive, maladaptive, or neutral to fitness. The changing pattern of a genotype with environment is usually characterized as a reaction norm. For continuous variables such as morphological, physiological, and life history variables, reaction norms are often visualized as a line or curve plotting the phenotypic value with the environmental value [1,2]. Variation in different responses to different environments among genotypes is referred to as an interaction between genotype and environment (G × E) and can be visualized as the reaction norms of multiple genotypes on a plot [2]. Thus, the evolution of genotypic plasticity can be visualized as a change in the slope of the reaction norm. Adaptive plasticity allows a genotype to have a broader tolerance to environmental changes. Such plasticity not only lessens extinction pressure in new environments, but also aids populations to move from one adaptive peak to another [2,3].

Phenotypic plasticity in zooplankton involves various morphological, behavioral, physiological, and life history responses that are not usually mutually exclusive. Changes in physiology and behavior involve the earlier timing of diapause to lessen fish predation [4], the accumulation of more protective pigments and/or strong avoidance via vertical migration [5] when exposed to ultraviolet radiation, and deeper distribution in response to predator cues and shallower distribution in response to hunger [6]. Each of these examples of phenotypic plasticity is triggered by top-down forces that have consequences for zooplankton survival. Another common type of plasticity is variable growth due to bottom-up forces. Food quantity and quality, such as elemental limitation (especially phosphate), digestion resistance, and biochemical limitation (especially fatty acids), can contribute to differences in growth rates [7]. At first appearance growth responses to diet may not have adaptive potential, but they can evolve with natural selection when different genotypes exhibit variable growth with food change [8].

Severe eutrophication in many freshwater systems causes frequent and prolonged cyanobacterial blooms [9]. Cyanobacteria are usually considered to be a “poor” food source for zooplankton. Mechanical interference, nutritional insufficiency, and toxicity are three main mechanisms by which cyanobacteria seriously affect zooplankton [10–12]. According to the arms-race hypothesis, zooplankton may develop counter-adaptations to beat the deleterious consequences of cyanobacteria. A short-time exposure to toxic cyanobacteria induces zooplankton defenses and enhances their fitness in cyanobacterial conditions [13–15]. These inducible defenses developed within one generation can be transferred to following generations via maternal effects and can contribute to neonatal success [16–19]. In addition to physiological adaptations, zooplankton can rapidly evolve genetically based resistance to toxic cyanobacteria [20–23].

Cladocerans are dominant zooplankton grazers in many freshwater ecosystems. One of their distinct characters is obligate parthenogenesis most or all of the time. As a consequence, their natural populations usually consist of several to many genetically different clones that coexist but respond differently to environmental changes [24]. Large clonal variation in the sensitivity to toxic cyanobacteria has been documented in daphniids. Daphniid clones isolated from eutrophic lakes are more resistant to toxic cyanobacteria than clones from oligotrophic ones [22]. Within a lake, clones born during eutrophication are more resistant to toxic cyanobacteria than clones born before eutrophication [20]. This increased resistance evolves as a decrease in phenotypic plasticity in which clones born during eutrophication increase the overall fitness of the population [8].

However, most previous studies on the interactions between cyanobacteria and zooplankton have focused on large cladocerans, mainly daphniids. Bosminids are small-size cladocerans, and are the dominant species in many lakes. Some studies have shown that bosminids differ from daphniids with respect to life history parameters [25], feeding [26], swimming behavior [27], sensitivity to copper stress [28]. The spatial distribution of bosminids ranges from cyanobacterial bloom to non-bloom areas and the seasonal distribution occurs during both bloom and non-bloom periods [29]. Bosminids appear to be less affected by toxic cyanobacteria than daphniids [30]. With the onset of cyanobacterial blooms, daphniid abundance decreases drastically, and this is associated with an apparent increase in bosminids [31]. In the present study, we ask the following questions: (1) Are there clonal variations in growth plasticity within a bosminid population when phytoplankton composition in summer shifts toward greater dominance by cyanobacteria? (2) If so, how do these changes in reaction norms contribute to the increased resistance of bosminids to toxic cyanobacteria?

## Materials and Methods

No specific permits were required for the described field works since the locations are not private-owned or protected in any way, and the zooplankton samplings did not involve endangered or protected species.

The microcystin-producing cyanobacterium *Microcystis* *aeruginosa* (FACHB-905) and the green alga *Chlorella pyrenoidosa* (FACHB-15) were obtained from the Freshwater Algae Culture Collection of the Institute of Hydrobiology, the Chinese Academy of Sciences. Algal cultures were incubated at 25°C under fluorescent lights at 50 µmol m^-2^ s^-1^ on a 12: 12 light : dark cycle. The FACHB-905 strain of *M* *. aeruginosa* grows as single or paired cells in laboratory conditions, which minimizes the potential effects on zooplankton due to mechanical interference. The carbon contents of *M* *. aeruginosa* and *C* *. pyrenoidosa* are 3.81 and 2.59 pg cell^-1^, respectively, estimated by their cell volumes.

Using a 60-µm mesh net, zooplankton samples were collected from Yingtao River near East China Normal University (E121.447^°^, N31.032^°^) on March 10, 2012. The randomly selected *Bosmina* *longirostris* adults were isolated from the samples and placed individually into 500 mL beakers to establish clones by parthenogenetic reproduction. The fifteen clones were isolated successfully and named with an arbitrary number (e.g. YT1, YT2, etc). Animals were provided *C* *. pyrenoidosa* daily at a carbon concentration of 400 µg C L^-1^ and half of the water was changed every three days. These clones were maintained for two months at 25°C in the laboratory to minimize both maternal effects and environmental variance prior to the direct comparison of clones.

A population growth experiment was conducted to evaluate *B* *. longirostris* performance when fed the “good” diet, *C* *. pyrenoidosa* , and the “poor” diet, *M* *. aeruginosa* , at the carbon concentration of 400 µg C L^-1^, with three replicates. Twenty *B* *. longirostris* with the age of 2 days (size class: 250-300 µm) were randomly transferred to a beaker containing 500 mL of algal suspension. Animals were reared for five days at 25°C with a daily renewal of half of algal suspensions. The number of live *B* *. longirostris* was counted under a microscope at the end of experiment. Population growth rate (*g*, day^-1^) was calculated using the following formulas: *g* = (lnN - ln20)/5, where N is the number of *B. longirostris* at day 5. The resistance (*R*) of each clone to dietary cyanobacteria was measured as the relative change in growth rates on the “poor” diet (*g*_poor_) compared with the “good” diet (*g*_good_): *R*= *g*_poor_/*g*_good_. This index was slightly modified from that used by Hairston et al. [20], but provides a more explicit indication of resistance [32]. The higher the index value is, the stronger the resistance of bosminids to toxic cyanobacteria.

The effects of food type and clone on growth rates of bosminids were tested using two-way ANOVA. A significant interaction between food type and clone would indicate that the difference in phenotypic plasticity in response to diets is genetically based. One-way ANOVA was used to compare resistance among clones. Data homogeneity and normality were confirmed by Leven’s test, Kolmogorov-Smirnov test, and Shapiron-Wilk test. Pearson correlation coefficient was used to investigate the correlation between two variables. All statistical tests were conducted using the Statistical Product and Service Solution (SPSS) 16.0 statistical package.

## Results

**Figure 1:**
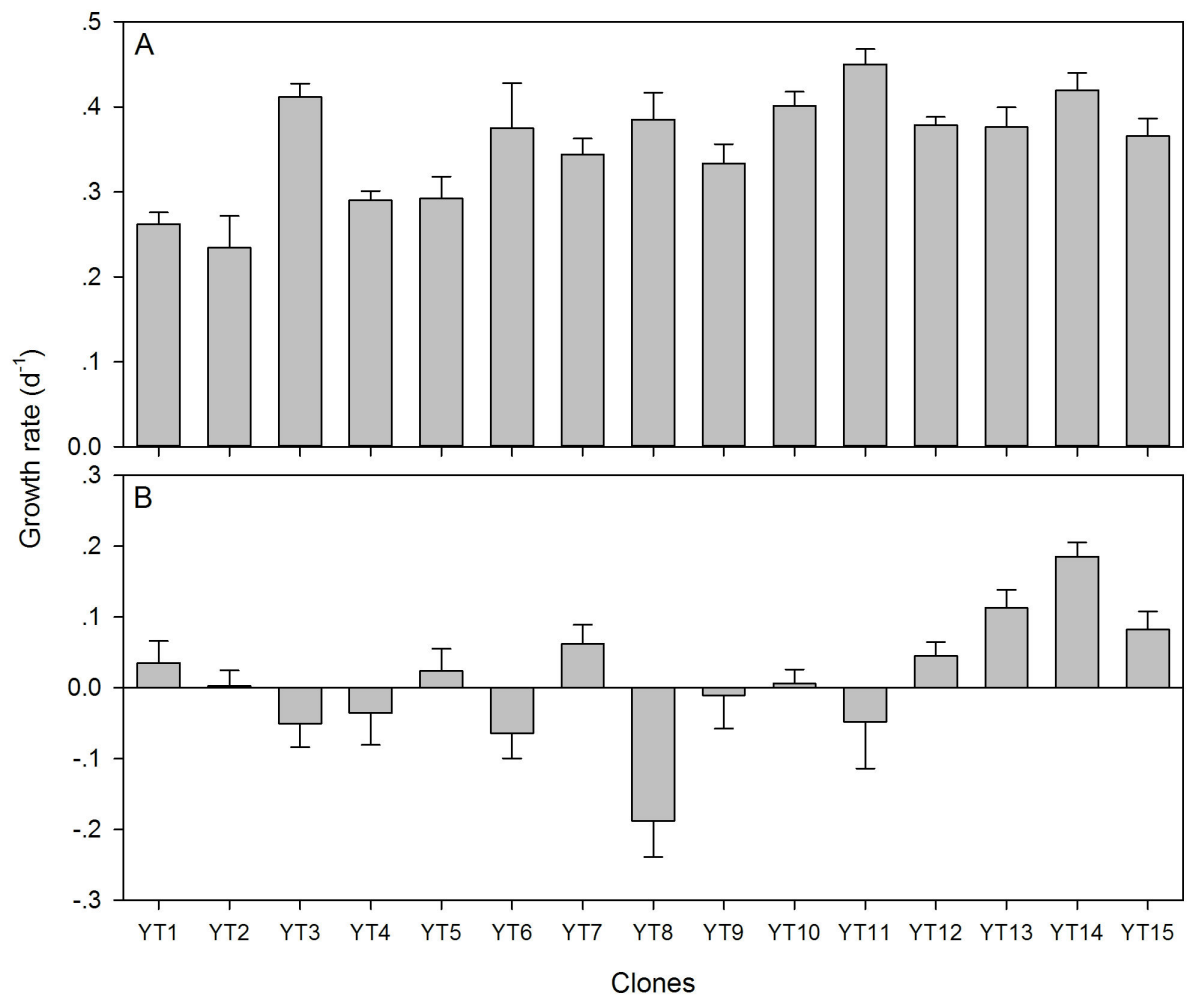
Population growth rates (means + SD) of 15 sympatric *Bosmina* *longirostris* clones feeding on (A) the “good” diets (*Chlorella pyrenoidosa*) and (B) the “poor” diets ( *Microcystis* *aeruginosa* ) at a carbon concentration of 400 µg C L^-1^.

**Figure 2:**
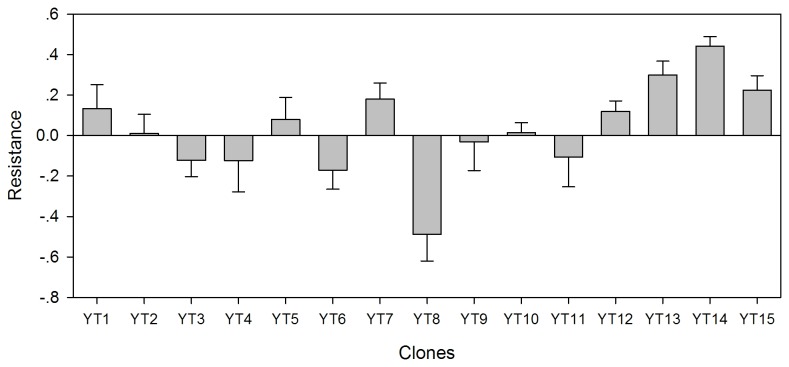
Resistance index (means + SD) of 15 sympatric *Bosmina* *longirostris* clones to toxic *Microcystis* *aeruginosa* .

Food type significantly affected *B* *. longirostris* growth (Two-way ANOVA, *F*_1,89_ = 2797, *P* < 0.001). The “good” diet, *C* *. pyrenoidosa* , supported positive growth in all clones of *B* *. longirostris* with *g*-values ranging from 0.23 to 0.45 day^-1^ (Figure 1A). The microcystin-producing *M* *. aeruginosa* proved to be a “poor” food source for *B* *. longirostris* with markedly low *g*-values, even negative values (Figure 1B). In addition to the effect of food type on the growth rate of *B* *. longirostris* , there was also a significant difference among clones (Two-way ANOVA, *F*_14,89_ = 17.60, *P* < 0.001). The significant clone × food-type interaction (Two-way ANOVA, *F*_14,89_ = 18.00, *P* < 0.001) indicated there were genetic differences in phenotypic plasticity for the response of growth rate to diet in *B* *. longirostris* . The resistance varied significantly among *B* *. longirostris* clones (One-way ANOVA, *F*_14,44_ = 14.42, *P* < 0.001). The clone YT14 was the most tolerant to *M* *. aeruginosa* , while the clone YT8 was the most sensitive one (Figure 2).

**Figure 3:**
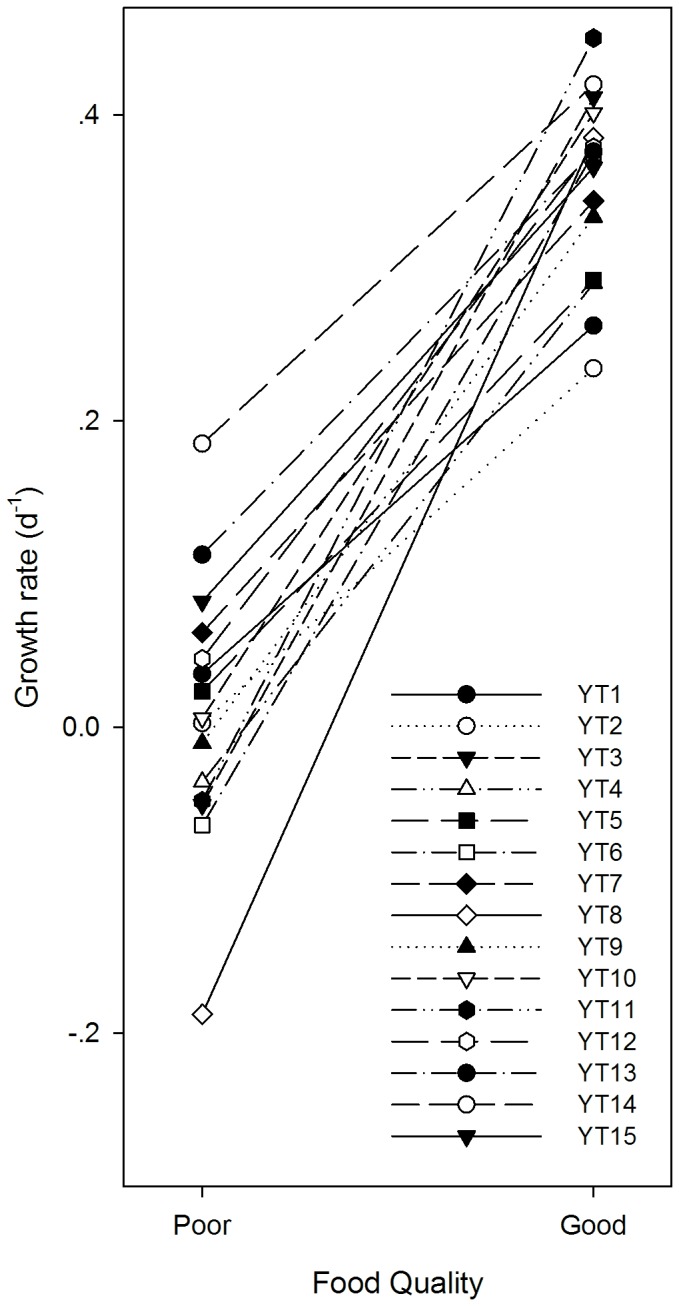
Reaction norms for growth rates of *Bosmina* *longirostris* feeding on the “poor” and “good” diets. Each line represents one of the 15 clones isolated from the same population.

**Figure 4:**
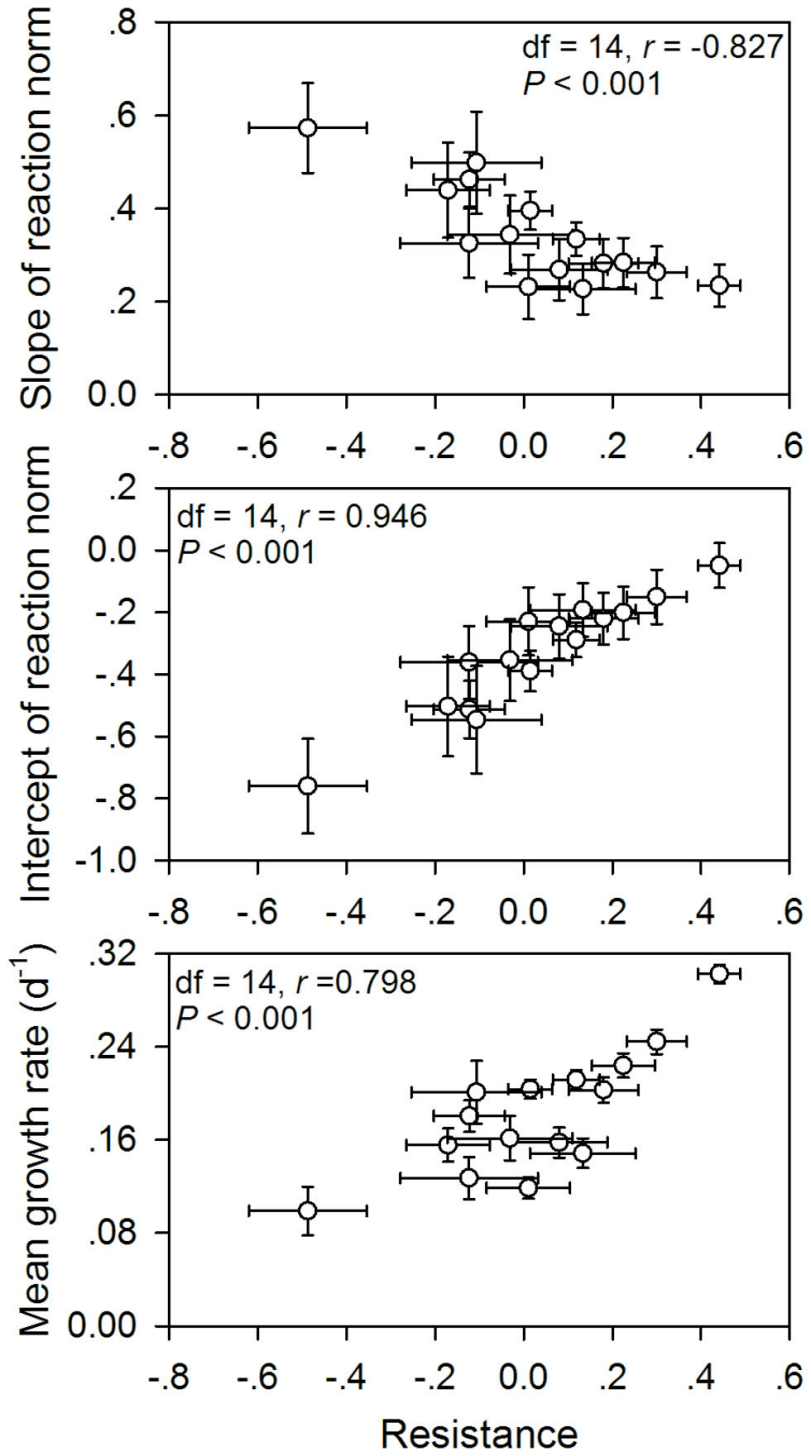
Correlations between the resistances to toxic *Microcystis* *aeruginosa* in *Bosmina* *longirostris* with three traits of reaction norms of phenotypic plasticity over two diets. Each data point represents one of the 15 sympatric clones.

The reaction norms of growth rates for each clone are shown in Figure 3. The slope and mean of the reaction norm were clone-specific (Figures 3 and 4). The resistance was negatively correlated with the slope (df = 14, Pearson correlation coefficient = -0.827, *P* < 0.001) and positively correlated with the intercept (df = 14, Pearson correlation coefficient = 0.946, *P* < 0.001) of the reaction norms (Figure 4). In addition, the resistance was positively correlated with the mean growth rate value (df = 14, Pearson correlation coefficient = 0.798, *P* < 0.001) of *B* *. longirostris* feeding on both diets (Figure 4).

**Figure 5:**
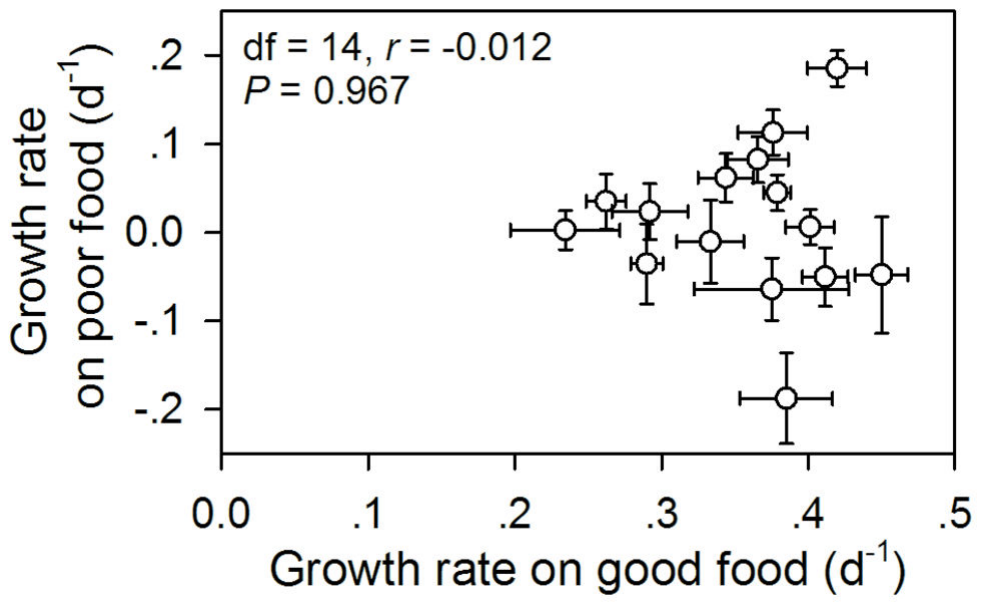
Growth rates of *Bosmina* *longirostris* when feeding on the “poor” diet versus that of individuals feeding on the “good” diet.

**Figure 6:**
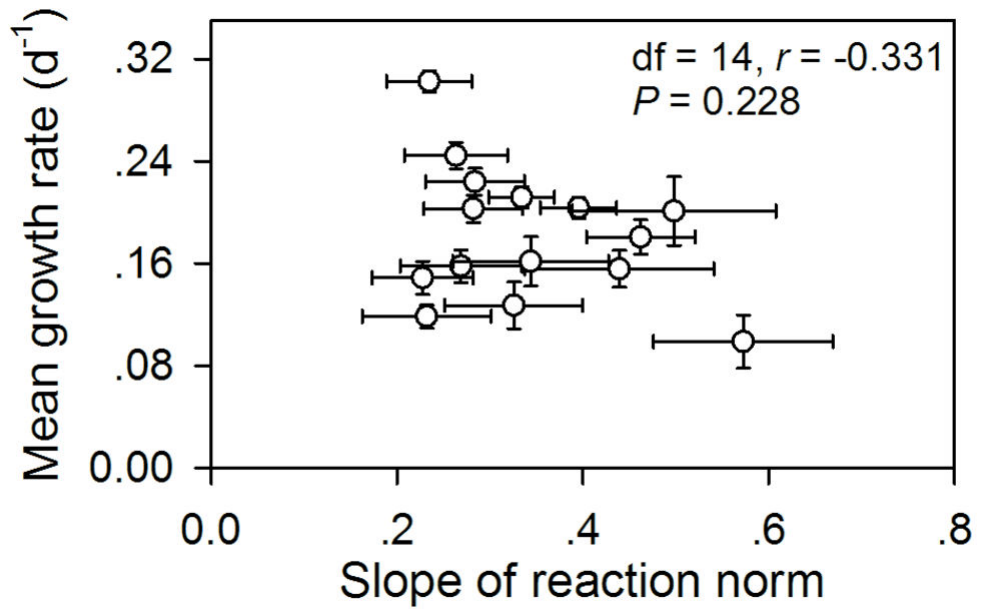
Slopes of reaction norms for 15 sympatric *Bosmina* *longirostris* clones versus the mean growth rates when feeding on the “good” and “poor” diets.

The growth rate of *B* *. longirostris* when fed the “good” diet was not significantly correlated with that of individuals on the “poor” diet (df = 14, Pearson correlation coefficient = -0.012, *P* = 0.967, Figure 5). There was also no significant correlation between the mean growth rate over two diets and the slope of the reaction norms (df = 14, Pearson correlation coefficient = -0.331, *P* = 0.228, Figure 6).

## Discussion

Phenotypic variability in morphological characters like body size and shape, antennule and mucro length in response to temperature and predation has been well documented in many bosminid species [33]. This phenomenon has been described as cyclomorphosis, temporal and cyclic morphological changes that occur within a planktonic population [27]. The present study provides strong evidence of the extensive growth variation in response to *M* *. aeruginosa* within a single population of *B* *. longirostris* . Since clones were incubated independently under uniform conditions, it can be assumed that significant differences in growth rate among clones would be genetically based rather than being simply inducible defenses. Our clones were derived from individual *B* *. longirostris* adults collected from the field. These clones were treated as genetically distinct in the present study, although genetic characterizations of each clone were not checked by molecular analyses. Thus, it is possible that two or more clones defined by microscopy, especially those with similar growth rates, may originate from the same clone. This potential limitation may result in the extent of clonal variation being underestimated, even though the clonal variation within a *B* *. longirostris* population is quite substantial.

This large variation in resistance to cyanobacteria in bosminids not only confirms previous studies reporting genetic differences in resistance among *Daphnia* clones from different populations [8,22,34,35], but also demonstrates that substantial clonal variation can occur within a population since our bosminid clones were randomly isolated from one zooplankton sample. Given that *M* *. aeruginosa* used in this study grows as single or paired cells, the clonal variation in *B* *. longirostris* does not include any differences in ability to deal with the morphological defenses of cyanobacteria. In the field, *M* *. aeruginosa* usually forms large colonies that depress zooplankton grazing and complicate interactions between cyanobacteria and zooplankton [36]. The clonal variation within a field population could be larger than the variation demonstrated by the present study. The mechanism for the large variation within a *B* *. longirostris* population is not clear. Cladocerans can form various clones by sexual reproduction. The frequency of rare favorable recombinants can be increased by clonal replication that reduces the possibility of their loss by genetic drift [37]. Clonal variations of cladocerans among and within populations suggest that the reliance of many previous laboratory studies on single clone may limit their applications to nature. Although we have demonstrated large clonal variation of phenotypic plasticity in *B* *. longirostris* , the present study cannot determine which clone represents an ancestral state from which other clones have evolved. Using techniques from resurrection ecology [38] and/or experimental evolution [39] may provide the answers to this unsolved question.

Large clonal variation in resistance to cyanobacteria within a bosminid population could serve as the fuel for local adaptation and subsequently contribute to a coevolutionary arms race between zooplankton and cyanobacteria. Increased cyanobacterial densities can act as an important selection agent on zooplankton populations since cyanobacteria produce harmful compounds [9]. In the context of exposure to toxic cyanobacteria, daphniids either induce resistance within a life-time and pass it on to following generations [16–19], or evolve rapidly resistance over multiple generations [20–23]. Rapid evolution of organisms in response to environmental changes usually stem from selection on pre-existing genetic variation because beneficial alleles are already available and their probabilities are often higher than de novo mutation [40]. Both the large variation in vulnerability to grazing within a single cyanobacterium [41] and the huge difference in sensitivity to toxic cyanobacteria within a single zooplankter would provide raw material for coevolution between cyanobacteria and zooplankton. Finally, since some clones have superior fitness in the presence of cyanobacteria, clonal succession with a single species may occur as well as species succession in the zooplankton community. Thus, cyanobacterial blooms, through their influence on the clonal composition of zooplankton populations, probably pose strong constraints on zooplankton responses to other environmental changes.

Not only does this study document intraspecific variation in resistance to cyanobacteria, but it also provides the first example of a negative relationship between the resistance and the slope of reaction norms. Hairston et al. [8] proposed two distinct mechanisms by which phenotypic plasticity evolves to enhance daphniid resistance against toxic cyanobacteria: a decrease in the slope of reaction norms showing a reduced sensitivity to cyanobacteria or an increase in the mean value over both “good” and “poor” diets. Both the negative correlation between the resistance and the slope of reaction norms and the positive correlation between the resistance and the intercept of reaction norms clearly suggest that the enhanced resistance to toxic cyanobacteria in *B* *. longirostris* , at least partly, is due to the evolution of reduced phenotypic plasticity, namely the decrease the slope of reaction norms. However, the positive correlation between the resistance and the mean of reaction norms over both “good” and “poor” diets suggests that an increase in the mean growth rate of *B* *. longirostris* feeding on both diets also contribute to the enhanced resistance to toxic cyanobacteria, which is consistent with a previous report on *D* *. galeata* [8]. Although the juvenile growth rates of *D* *. galeata* on a “poor” diet are significantly correlated with those on a “good” diet [8], there is no evidence for this correlation in *B* *. longirostris* . Thus, two distinct mechanisms for evolution of phenotypic plasticity may be not mutually exclusive. Both mechanisms may work simultaneously to enhance zooplankton resistance against toxic cyanobacteria via reducing phenotypic plasticity. The correlation between the slope and the mean of the reaction norms is one of the important parameters for evaluating heritability measurements and evolutionary dynamics [1]. The slope of reaction norms evolves independently of or jointly with the mean trait value [2]. Some studies have shown that the correlation coefficients ranged from 0 to 1 with a median value of 0.41 [1]. Our results suggest that the slope and the mean of reaction norms may evolve as separate traits in *B* *. longirostris* since their correlation was not significant.

Phenotypic plasticity provides the potential for organisms to respond rapidly and effectively to environmental changes. It can, therefore, play a central role in tracking environmental changes [1,2]. The present study shows that *B* *. longirostris* , a cosmopolitan and dominant zooplankter in eutrophic waters, responds through a simple change in growth rate to the presence of cyanobacteria in their diets. In addition to significant food and clonal effects on *B* *. longirostris* growth rates, the significant clone × food-type interaction indicates genetic differences in phenotypic plasticity. Both the reduced slope and the increased mean of reaction norms contribute to the evolution of enhanced resistance against cyanobacteria in *B* *. longirostris* . Since *B* *. longirostris* clones differ genetically in the magnitude and pattern of their responses, natural selection may drive evolutionary change in plasticity. Large clonal variation in resistance to cyanobacteria and its ecological consequences may influence the clonal composition of cladoceran populations. The occurrence and development of cyanobacterial blooms may provoke simultaneously clonal and species succession in the zooplankton community, and subsequently shift the functions of aquatic ecosystems.
